# Seventh Annual Session of the Maryland State Dental Association

**Published:** 1890-08

**Authors:** 


(To be continued.)

---

### SEVENTH ANNUAL SESSION OF THE MARYLAND STATE DENTAL ASSOCIATION.

(Continued from page 420.)

*Friday, December 6, 1889.—Morning Session.*

Report of Committee on Operative Dentistry, by C. M. Gingrich, D.D.S., Baltimore.

There is perhaps no subject of so much interest and importance to the practitioner of dentistry, and no department in our profession in which there is so much *general interest* as that of Operative Dentistry. To this department, more especially than to any other in the profession, devolves the preservation of the natural teeth, and it is the desire of your committee that this subject receive from the association the attention commensurate to the importance of the interests involved.

The standard to which the profession has attained is the result of scientific and intelligent investigation, indefatigable exertion, and unremitting labor. The men who, fifty years ago, labored so faithfully, who were so hopeful, so sanguine, and who expected so much for the future of the young profession, never dreamed that in so short a time so much would have been accomplished.

The profession of to-day is beginning to properly appreciate and understand the scientific and intelligent application of all that modern research, investigation, and experience has developed.

The resources to which the operator in this day of progress has access are so unlimited, the means at his command so ample, that there can scarcely be an excuse to be confined within the narrow limits of a single theory which is made to subserve all indications.

The careful student of dental literature, and close observer of discussions in our dental societies, will notice that a change of sentiment is manifest and has been pervading the department of operative dentistry during the past decade, and one of the most healthful indications is the spirit of liberality and exercise of more discretion in the use of the different materials at our command for the preservation of the teeth of our patients.

Older operators remember when an attack was made on amalgam by some of the writers and exponents of operative dentistry who urged every possible argument against it, and even went so far as to have members expelled from dental societies who would not pledge themselves to discontinue the use of amalgam fillings. To-day, the profession is willing to use any material, though it be amalgam, when its use is indicated to preserve a valuable molar, and unprejudiced views now allow common sense to suggest this material when the success of a gold filling would be questionable.

The introduction of cohesive gold opened a new era in the practice of operative dentistry,—in fact, the induction of this new form of gold revolutionized practice in all that it implies and comprehends. Nothing has ever been given the profession which was so universally accepted and which met with so much general favor. The profession was almost a unit in its abandonment of non-cohesive and substituting therefore cohesive gold. It very soon became the theme of discussion in dental societies; the essayist needed no other text to entice the ear of his audience; the clinical demonstrator required no other mode, and the teacher gloried in the day of its acceptance. The cohesive era developed many forms of gold, including every gradation of heavy and light foils, together with crystal sponge and fibrous gold, cohesive and extra-cohesive, so that every imagination of the operator could be gratified. With it, also, came the various forms of mallets, those most generally accepted and in use being the electro-magnetic, automatic, and the hand-mallet, all of which have their advocates and individual preferences.

The question may very properly be asked, Have the advocates of cohesive gold realized all that was expected? Have the results been as gratifying as the promises made? Have they performed better operations than their predecessors, and saved as many teeth in proportion to the number filled as those who knew nothing of cohesive gold? In the first place, we answer we do not believe that cohesive gold offers the advantage in a great majority of cases as a filling material that "the old fashioned" foil does.

In difficult and remote cavities, the operator to use this gold is compelled to do much unnecessary cutting away very often of valuable tooth structure, so as to make the cavity accessible.

It is conceded that almost direct access must be obtained in order to use this foil to even approximate ordinary adaptation.

On the other hand, non-cohesive gold, yielding readily under the pressure of the instrument, can be driven to the remotest parts of the cavity, and perfect adaptation secured with half the effort on the part of the operator and less discomfort to the patient.

Let us take for illustration ordinary cavities in the six anterior superior teeth. How can the operator justify himself in cutting these teeth through from the labial surface in order to gain access simply to fill the cavities with cohesive gold? Again, how can he justify himself to display gold in a patient's mouth when it could have been so easily avoided? Every operator with any experience has seen these operations done as here briefly described.

With non-cohesive gold these cavities can be as conveniently reached by cutting the teeth from the palatal surface and entirely exposing the cavities and leaving the labial surface intact, thus preserving the beauty of the teeth, not destroying half as much tooth structure, and preserving the teeth as well. We assert, and we do not believe there is a member in this association who will have the assurance to say that there were ever better operations and more teeth saved than by the old operators, who were confined to the exclusive use of non-cohesive gold. It is a genuine pleasure and gratification to see these operations that have stood untarnished for more than a quarter of a century. They stand as a monument perpetuating the memory and ability and mode of practice of the men who laid the corner-stone of our noble calling.

As an adjunct to operative dentistry, nothing has ever been given to the profession of so much value to both operator and patient as cohesive gold. In the restoration of contour, when it is indicated and required, nothing can take its place, and we have nothing at our command to even approximate its usefulness.

To replace broken-down tooth structure it is the only resource at our command, and, as intimated above, it is an invaluable auxiliary in operative dentistry, and it is not probable that the profession will in the near future have control of any material with which it can so conveniently and so efficiently accomplish this most desirable and, very often, this most important part of an operation.

Neither the profession nor an individual can ever be justified in accepting any theory, mode, or material as a dogma, and make every condition in practice subservient to it. The faithful operator will always yield to judgment and discretion in the selection of material in order to bestow upon his patient the greatest good, whether it be non-cohesive or cohesive gold or any other material at his command. It is this very point we wish to emphasize, and we are glad to report to this society that the profession is rapidly extricating itself from the depth of narrow channels, hobbies, and dogmas, and placing its feet upon the rock of truth, where it will reflect its greatness on all future generations.

In illustration of this feeling in the profession, we recall the electro-chemical theory, or "new departure," as it was called a few years ago, which had for its text, "in proportion as teeth need filling, gold is the worst material to use." A few enthusiasts heralded this heresy from the Atlantic to the Pacific and urged every possible means to arbitrarily drive the profession to accept the dogma, which had no other merit than the escape of labor entailed by inserting an honest and permanent filling in a tooth which needed the very best material for its preservation.

After this came another from across the deep blue sea, which had nothing more to recommend it than the beautiful colors radiating from an uncrystallized piece of quartz. The profession, as in duty bound, took due notice of these new theories, and, upon investigation, relegated them to that oblivion to which they justly belonged.

Your committee is most happy to report progress in the profession, and that it is marching onward with gigantic strides.

Let us ever remember that "knowledge is power," and that on our intelligence rests the future prosperity, grandeur, and glory of our profession. Let us, then, go on with a laudable ambition and unyielding perseverance in the path which leads to honor and renown. Let us press forward and gather laurels from the hill of science, linger among her unfading beauties, "drink deep" of her crystal fountain, and we shall join the march of fame.

Respectfully submitted.

DISCUSSION OF DR. GINGRICH'S REPORT ON OPERATIVE DENTISTRY.

*Dr. E. Nelson*, Baltimore.—I listened to the reading of the paper by Dr. Gingrich with much care and interest. I very cordially endorse many of the positions which that gentleman assumed in the paper. If I mistake not, the essay does not unqualifiedly endorse soft gold as proper to be used in all cases,—that is my recollection of it,—but it assumes the position that, in the selection of material, or of the particular form of gold, for filling the teeth, the operator must be guided by his best judgment. The gentleman very properly alluded to the number of teeth that had been filled prior to the introduction of cohesive gold and which still remained as standing monuments of the skill and success of the operators by whom the fillings were inserted. I wish to corroborate the truthfulness of what he has said on that point by what has come under my own personal observation. I refer to the case of a sister of mine who is now residing in the city of Baltimore, for whom, thirty years ago, Dr. Volck inserted two fillings in the incisors, and I say to Dr. Volck that those fillings are to-day as good as they were on the day she left his office.

I agree with the author of the paper that there are circumstances in which soft gold is infinitely better than cohesive gold, and in which cohesive gold cannot be used. On the other hand, there are circumstances, owing to the position of the teeth, the location of the cavity, and the convenient use of the mallet, in which cohesive gold is of superior value. I think it is a mistaken idea for any one to confine himself exclusively to the use of any special form of gold, or to assert that, in all cases without exception, a particular form of gold is the only one to be used. It strikes me that the exercise of proper discretion and judgment in all cases, as to the form of gold, is the rule to be observed.

*Dr. Charles D. Cook*, Brooklyn, N. Y.—I did not have the privilege of hearing the paper read, as I was not present at the time, but as the subject of the use of what is known as non-cohesive or soft gold is to some extent under discussion, I may say that I probably belong to the past generation in the matter of the use of soft gold. That was the only form of gold of which we had knowledge when I commenced practice. Practically I have used it more or less from the day it was first introduced down to the present time, and I could not well dispense with it. If I were compelled to relinquish the use of either, I would prefer to part with crystal or cohesive gold, though, of course, I would do so reluctantly.

I have been in the habit of using the soft foil or non-cohesive foil at the cervical wall of approximal cavities and crystal gold, as the indications may seem to warrant, in connection with the soft foil. I use the soft foil first and then finish with the cohesive gold. I very seldom fill a cavity with cohesive gold alone. Crown cavities of molars can be filled with soft gold equally as well as with cohesive gold and much more quickly than with the latter, saving much time to the dentist and to the patient and presenting an equally good operation. I do not depend upon the union of the two for their retention in the cavity but upon the shape of the cavity, as though practically soft foil was used independently of the cohesive foil. As fine operations as any I have ever seen with any kind of foil were from the hands of Dr. Dunning, of New York City, one of the most accomplished operators we have ever had in America, who, as you know, has been prominent for his advocacy of the use of soft foil.

I think that our dental colleges have been negligent in failing to impart practical (not theoretical) instruction in the use of soft foil with the hand instrument. I think we are able to obtain results that are infinitely better than would be obtained by the exclusive use of cohesive foil. I do not see how the profession could well serve the public if soft foil was excluded from our practice.

*Dr. C. C. Harris*, Baltimore.—I have always tried to be an observer, and it was, I think, from observation that I acquired certain convictions. One of which is that in the practice of dentistry we must be conservative, and, first of all, we must utilize everything that is of value. We must learn to use both cohesive and non-cohesive gold. It strikes me that any one who uses both with a comparative degree of success will never be willing to give up one for the other. They must both be used together. I feel that if I was compelled to abandon the use of cohesive gold I would actually have to relinquish my practice. I do appreciate cohesive foil, and I realize that it is a good thing in its place and that there is nothing that can fully take its place. I have read much on the subject in recent years, and have observed that there are many advocates of the use of amalgam for the cervical walls and of the use of cohesive foil as you come to the grinding surface. That is where soft foil belongs, and, in the hands of a capable operator, it can be used there as successfully as amalgam. Amalgam possesses no advantage over soft foil for the cervical walls of the teeth. In that particular, I think, there is a tendency towards neglect in our work. When it is said by some men that the cervical walls fail more rapidly than other portions, it is quite likely that that is true; but if a man will keep his mind on his work and adapt his soft foil to the cervical wall thoroughly, if he will see that it is condensed there as permanently and as nicely as he wants to have it in other positions, I think he will eventually meet with success.

Only a short time ago I had two patients in my office, for whom I was working during the same month, one was a lady for whom Dr. Chapin A. Harris filled from ten to fourteen teeth with soft foil sixteen years ago. Those fillings were all standing and impervious. The other patient was a lady who had been treated eight or ten years previously by a prominent dentist. She had molars that were almost entirely built up of gold, and bicuspids with their approximal walls also contoured beautifully with cohesive gold. I had never seen more work in a single mouth than I saw in the mouth of that lady, but, with hardly an exception, her back teeth had all been patched with amalgam. I mention this as showing that cohesive foil from the best hands must fail if the other fails. Those cases presented two samples of the work of two of the best workmen in the country.

In connection with what I have said I want to mention that where gold and amalgam were both together, although the work was done years ago, the saving of the teeth from that time was, as far as I could judge, more apparent than it was with respect to the time prior to that.

I am inclined to believe that there is something in the talk which I have heard, to the effect that, where decay attacks the surface of a tooth around the gold filling and it is overcome with amalgam, the two will probably do better service than would be done by either one of them alone. At the same time, I do not yield on that point my opinion that soft foil, if inserted in those places which are more susceptible to attack from decay, will accomplish all that *can be claimed* for amalgam.

Some allusion was made in the essay to amalgam, and that leads me to say that there are many operators who seem to be ashamed to admit that they use amalgam. For my own part, I have to say that I am not ashamed to make the admission, but, on the contrary, I am proud to have it known that I do use amalgam. I believe that amalgam in its place is a good thing for both the rich and the poor. It is a desirable thing for rich people in that it saves them from the annoyance of prolonged and tedious operations which many of them perhaps could not endure, and for our poorer patients because it enables them to save their teeth by less expensive operations than they otherwise could have.

*Dr. Mills.*—Dr. Harris has spoken of some cases in which gold foil has lasted for a certain number of years. I am familiar with a case which, I think, is a little more remarkable than any to which allusion has been made. Recently I undertook to extract a tooth from the mouth of an aunt who was eighty-eight years of age. In the crown of that tooth was a filling of soft foil which had been placed there, as she informed me, exactly sixty years before. She stated that it had been made by Dr. Maynard, an old practitioner, of the city of Washington.

I will say that I am an advocate of the use of soft foil in some cases and of cohesive foil in others. I think the use of the two should be combined, and that, as Dr. Harris has suggested, we should not assume a radical or ultra position by contending for the exclusive use of either.

In connection with what has been said, I may add that sometimes my fillings of oxyphosphate of zinc remain perfect, and I have tried to ascertain what it is that makes them do so under certain conditions and not under others. There was one case in which a lady brought her daughter to my office and stated that she wished to have her teeth filled with gold, but I refused to comply with her request because of the soft condition of the patient's teeth. I was allowed to have my own way about it, and I filled the teeth with oxyphosphate of zinc. The fillings I inserted remained for two years, and at the expiration of that time showed very little disintegration. Believing the teeth to be then in a condition to warrant it, I filled them with gold. Upon sending for the patient within six months thereafter and having made an examination of the mouth, I discovered that, of these latter fillings which I had inserted, five had dropped out and only one remained, and that one was rolling around in the cavity. I again filled her teeth with oxyphosphate of zinc. I remember that I filled two bicuspids with oxyphosphate of zinc from the same mix, when it was in that state which we call "crystallization," or beginning to crumble. I kneaded it together into a soft paste between my thumb and finger and forced it into the cavities rather carelessly and more for the purpose of experimentation than anything else. Much to my surprise, two years or more after, I discovered that the fillings were not only remaining perfect and as they were when inserted, but that the surfaces had assumed a hard, glassy appearance.

